# Ring Contracting Sulfur Extrusion from Oxidized Phenothiazine Ring Systems

**DOI:** 10.3390/molecules13061345

**Published:** 2008-06-12

**Authors:** Steven C. Farmer, Seth H. Berg

**Affiliations:** Department of Chemistry, Sonoma State University, 1801 East Cotati Avenue, Rohnert Park, California 94928, USA

**Keywords:** Phenothiazine, Desulfurization, Sulfoxides, Sulfones

## Abstract

Lithium, used in conjunction with sodium metal, produces a high yield of carbazole when reacted with phenothiazine-5-oxide or phenothiazine-5,5-dioxide. A high yield of 9-ethylcarbazole is also produced when these reagents react with 10-ethyl-phenothiazine, 10-ethylphenothiazine-5-oxide, and 10-ethylphenothiazine-5,5-dioxide. Degassed Raney nickel produces carbazole in high yield when reacted with phenothiazine and phenothiazine-5-oxide. A moderate yield of 9-ethylcarbazole is produced when *n*‑butyllithium is reacted with 10-ethylphenothiazine-5-oxide.

## Introduction

Past research has focused on the sulfur extrusion reactions of heterocycles [1], mainly because of their applications in the desulfurization of fossil fuels [2]. Some examples of the ring contraction of phenothiazine (**1**) to make carbazole (**2**) and of 10-ethylphenothiazine (**3**) to make 9-ethylcarbazole (**4**) [3,4,5] have been reported. However, to our knowledge there have been no reported attempts at causing a similar desulfurization from the phenothiazine-5-oxide (**5**), phenothiazine-5,5-dioxide (**6**), 10-ethyl-phenothiazine-5-oxide (**7**) and 10-ethylphenothiazine-5,5-dioxide (**8**) as S-oxidized derivatives of this ring system. An efficient method for these ring contractions could prove useful because most synthesis routes to derivatives of phenothiazine [6] could then be directly applied to the corresponding carbazoles. In this work we apply our previous work using lithium and sodium [7], Badger’s work with degassed Raney nickel [8] and Gilman's work with *n*-butyllithium [9] to provide the first reported ring contracting desulfurization of phenothiazine-5-oxides or phenothiazine-5,5-dioxides.

## Results and Discussion

**Scheme 1:**
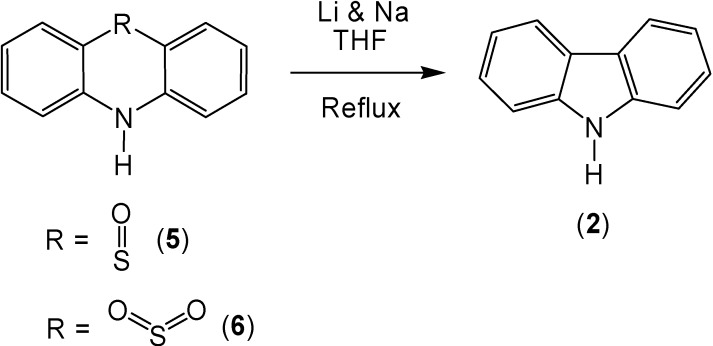


Previously, it has been shown that lithium, used in conjunction with sodium metal produces a high yield of carbazole (**2**) when reacted with phenothiazine (**1**) [7]. Owing to the fact that the mechanism of this reaction involves the formation of an anion, it was anticipated that the sulfoxide and sulfone derivatives of phenothiazine could stabilize the intermediates of this reaction; thereby, increasing the carbazole yield (Scheme 1). This turned out to be the case with phenothiazine-5-oxide (**5**), which produced a 96% yield of carbazole (**2**). However, the use of phenothiazine-5,5-dioxide (**6**) produced almost no increase in the carbazole yield (90%).

**Scheme 2:**
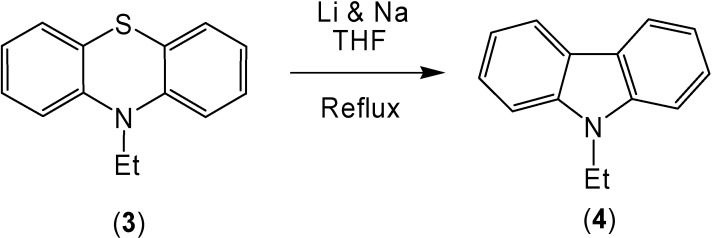


**Scheme 3:**
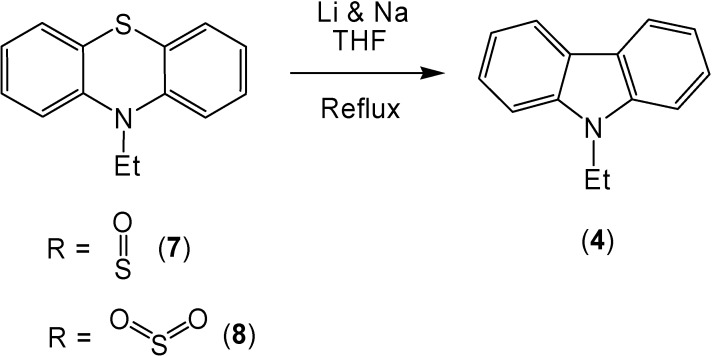


The combination of lithium and sodium also caused the conversion of 10-ethylphenothiazine (**3**) to 9-ethylcarbazole (**4**) in 86% yield (Scheme 2). The oxidation of the sulfur in the 10-ethylphenothiazine ring system caused an increase of the corresponding ring contracted product (Scheme 3). After reaction, 10-ethylphenothiazine-5-oxide (**7**) produced 9-ethylcarbazole (**4**) in a 98% yield and 10-ethyl-phenothiazine-5,5-dioxide (**8**) produced a 94% yield of 9-ethylcarbazole (**4**).

Reacting thianthrene with degassed Raney nickel produces a small yield of dibenzothiophene [8]. It was assumed that the low yield of a ring contracted product was caused by the presence of two sulfur atoms, both of which can be extruded. The use of Raney nickel on the phenothiazine ring system has been shown to form diphenylamine [10]. However, using degassed Raney nickel changes the product of the reaction to carbazole, because there is little absorbed hydrogen to react with the free radicals produced by this process.

**Scheme 4:**
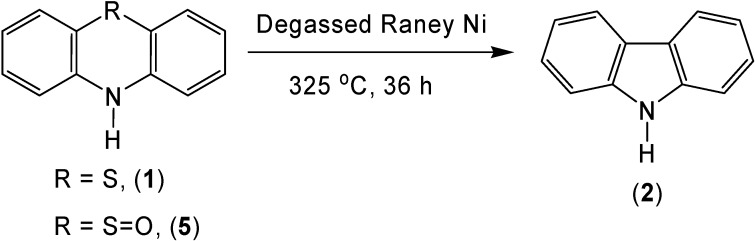


As expected, the use of degassed Raney nickel allowed for the generation of a high yield of carbazole (**2**) (69 %) when it was reacted with phenothiazine (**1**) (Scheme 4). Due to the mechanism for this reaction, it was theorized that oxidized sulfur might offer a better leaving group allowing for a higher carbazole yield or a decrease in the reaction temperature. Although sulfur extrusion with phenothiazine-5-oxide (**5**) did occur, no significant increase in yield (72 %) or decrease in reaction temperature was seen.

**Scheme 5:**
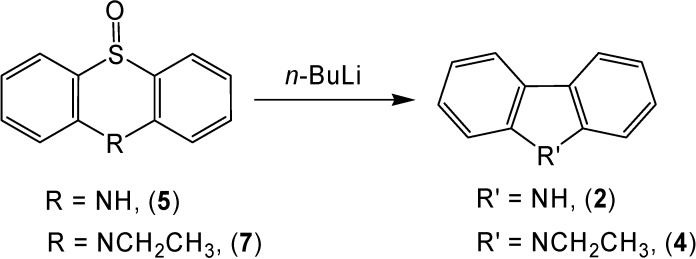


Gilman [9] used *n*-butyllithium to perform a sulfur extrusion from thianthrene-5-oxide to provide a moderate yield of dibenzothiophene. The mechanism for this reaction involves nucleophilic attack on the sulfoxide moiety. The corresponding reaction of phenothiazine-5-oxide (**5**) with *n*-butyllithium only produced a small yield (15 %) of carbazole (**2**), presumably due to the presence of an acidic hydrogen on nitrogen (Scheme 5). An idea to rectify this problem was to protect the nitrogen with an alkyl group. A repeat of the reaction with 10-ethylphenothiazine-5-oxide (**7**) produced a moderate yield (47 %) of 9-ethylcarbazole (**4**).

## Conclusions

By providing efficient, high yield methods for the ring contraction of oxidized phenothiazine ring systems we have opened more possible synthesis routes for carbazole derivatives. Also these improvements may now be applied to other types of compounds that contain the thiazine ring system.

## Experimental

### General

All IR spectra were recorded neat on a NaCl plate on a Mattson Galaxy Series FTIR 3000 infrared spectrometer. ^1^H- and ^13^C-NMR were obtained in CDCl_3_ on a General Electric QE-300 at 300 MHz and 75 MHz, respectively, using TMS as the reference. All GCMS spectra were recorded using a Varian 3400 Gas Chromatograph and a Finnigan MAT ion trap detector. All reactions were performed under a nitrogen atmosphere using oven-dried glassware. Melting points are uncorrected and were measured using a Thomas Hoover capillary melting point apparatus. Chromatography was accomplished using 20 to 230 mesh silica gel. Thin layer chromatography was performed using plastic backed silicagel 60 F_254_ plates. Unless otherwise stated, all reagents were obtained from commercial sources and were used without further purification. The compounds 10-ethyl-phenothiazine-5-oxide (**7**) [11], phenothiazine-5-oxide (**5**) [11], and phenothiazine-5,5-dioxide (**6**) [12] were all made using literature methods.

### Carbazole *(**2**)* from phenothiazine-5-oxide *(**5**)* using Li/Na

Phenothiazine-5-oxide (**5**, 0.100 g, 0.465 mmol) was dissolved in THF (40 mL) in a 100 mL round-bottomed flask. To this was added Li dispersion (27.7 mg, 4.00 mmol) and Na metal (92.0 mg, 4.00 mmol). The mixture was heated at reflux for 24 h, after which time any remaining Li or Na was destroyed with careful addition of MeOH. Work-up consisted of washing the reaction mixture twice with 5% NaOH (75 mL) and once with water (75 mL). The aqueous layers were combined and back-extracted with THF (75 mL). The combined organic layers were dried with sodium sulfate, filtered and evaporated to produce a white powder which was purified on a silica column, using a 1:3:16 mixture of MeOH, CH_3_Cl and petroleum ether as the eluent to give 73.8 mg (0.441 mmol, 95% yield) of pure carbazole (**2**); m.p. 242-244 °C (lit. m.p. 240-243 °C [13]); IR (cm^-1^): 3420 (N-H); ^1^H-NMR δ (ppm) [13]: 7.20 (dd, 1H, *J* = 7.7 Hz, 7.2 Hz), 7.39 (dd, 1H, *J* = 8.0 Hz, 7.2 Hz), 7.51 (d, 1H, *J* = 8.0 Hz), 8.11 (d, 1H, *J* = 7.7 Hz); ^13^C-NMR δ (ppm) [13]: 110.8, 118.6, 120.0, 122.7, 125.2, 139.6; MS (EI, m/z) 167 (M^+^).

### Carbazole *(**2**)* from phenothiazine-5,5-dioxide *(**6**)* using Li/Na

The same general procedure was followed as above except Na metal (0.173 g, 7.50 mmol) and Li dispersion (52.1 mg, 7.50 mmol) were reacted with phenothiazine-5,5-dioxide (**6**, 0.173 grams, 0.750 mmol). The reaction produced 112.7 mg (0.675 mmole, 90% yield) of pure carbazole; m.p. 242-243°C; IR and NMR identical in all respects to an authentic sample.

### 9-Ethylcarbazole *(**4**)* from 10-ethylphenothiazine *(**3**)* using Li/Na

The same general procedure was followed as above except Na metal (0.102 g, 4.40 mmol) and Li dispersion (30.6 mg, 4.40 mmol) were reacted with 10-ethylphenothiazine (**3**, 0.100 grams, 0.440 mmol). The reaction produced 73.9 mg (0.379 mmole, 86% yield) of pure 9-ethylcarbazole (**4**); m.p. 67-68 °C (lit. m.p. 63-66 °C [3b]); IR (cm^-1^): 3050 (C-H); ^1^H-NMR δ (ppm) [14]: 1.45 (t, 3H, *J* = 5.0 Hz), 4.40 (q, 2H, *J* = 7.0 Hz), 7.30 (dd, 2H, *J* = 7.6 Hz, 7.2 Hz), 7.48 (d, 2H, *J* = 7.9 Hz), 7.52 (dd, 2H, *J* = 7.9 Hz, 7.2 Hz), 8.18 (d, 2H, *J* = 7.6 Hz); ^13^C-NMR δ (ppm) [15]: 14.2, 38.1, 109.2, 119.6, 120.0, 122.8, 126.2, 140.5; MS (EI, m/z) 195 (M^+^).

### 9-Ethylcarbazole *(**4**)* from 10-ethylphenothiazine-5-oxide *(**3**)* using Li/Na

The same general procedure was followed as above except Na metal (94.8 mg, 4.11 mmol) and Li dispersion (28.6 mg, 4.11 mmol) were reacted with 10-ethylphenothiazine-5-oxide (**3**, 0.100 grams, 0.411 mmol). The reaction produced 78.7 mg (0.404 mmole, 98% yield) of pure 9-ethylcarbazole (**4**); m.p. 67-68 °C; IR and NMR identical in all respects to an authentic sample.

### 9-Ethylcarbazole *(**4**)* from 10-ethylphenothiazine-5,5-dioxide *(**3**)* using Li/Na

The same general procedure was followed as above except Na metal (89.0 mg, 3.86 mmol) and Li dispersion (26.8 mg, 3.86 mmol) were reacted with 10-ethylphenothiazine-5,5-dioxide (**3**, 0.100 grams, 0.386 mmol). The reaction produced 70.6 mg (0.362 mmole, 94% yield) of pure 9-ethyl-carbazole (**4**); m.p. 67-68 °C; IR and NMR identical in all respects to an authentic sample.

### Carbazole *(**2**)* from phenothiazine *(**1**)* using degassed Raney Ni

W-2 Raney nickel (1.35 g) was placed in a two-necked 150 mL round-bottomed flask. One neck was attached to a vacuum pump, while the other was fitted with a reflux condenser, topped with a capped addition funnel filled with mineral oil (40 mL). The Raney nickel was heated under vacuum to 250 °C, for 2 h. [*Caution!* Degassed Raney nickel is explosive!]. After this time, the mineral oil was added, the addition funnel removed, the reflux condenser and the second neck was capped and the reaction was continued under atmospheric pressure. Phenothiazine (**1**, 0.150 g, 0.753 mmol) was added and the mixture was heated to 325 °C. After 48 h the reaction was complete and the reaction flask was cooled. The mineral oil was removed with a silica column using petroleum ether as the eluent, then the remaining carbazole was eluted using 4% MeOH in CH_2_Cl_2_. Further purification consisted of a silica column, using a 1:3:16 mixture of MeOH, CH_3_Cl and petroleum ether as the elution solvent, followed by recrystallization from EtOH to produce 86.8 mg (0.520 mmol, 69% yield) of carbazole (**2**) as white crystals; m.p. 241-243°C; IR and NMR identical in all respects to an authentic sample.

### Carbazole *(**2**)* from phenothiazine-5-oxide *(**5**)* using degassed Raney Ni

The same general procedure was followed as above except W-2 Raney nickel (1.35 g) was reacted with phenothiazine-5-oxide (**5**, 0.173 grams, 0.750 mmol) [11] to afford after purification 83.8 mg (0.502 mmol, 72% yield) of carbazole (**2**); m.p. 241-243°C; IR and NMR identical in all respects to an authentic sample.

### Carbazole *(**2**)* from phenothiazine-5-oxide *(**5**)* using n-butyllithium

Phenothiazine-5-oxide (**5**, 0.300 g, 1.39 mmol) [11] was placed in a 25 mL round-bottomed flask with THF (10 mL). The flask was placed under a nitrogen atmosphere, then cooled to -78 °C in a dry ice / acetone bath. At this time 1.6 M *n*-butyllithium (0.383 mL, 0.268 g, 4.18 mmol) was added via syringe. The reaction mixture was stirred for 7 h, warmed to room temperature and finally heated at reflux for 24 h, after which time any remaining *n*-butyllithium was destroyed by careful addition of MeOH. Work-up consisted of washing the reaction mixture twice with 5% NaOH (20 mL) and once with water (20 mL). The aqueous layers were combined and back-extracted with THF (20 mL). The organic layers were dried with sodium sulfate, filtered and evaporated to produce a white powder. Purification consisted of a silica column, using a 1:3:16 mixture of MeOH, CH_3_Cl and petroleum ether as the elution solvent, followed by recrystallization from EtOH to produce 34.7 mg (0.208 mmole, 15% yield) of pure carbazole (**2**) as white crystals; m.p. 242-243°C; IR and NMR identical in all respects to an authentic sample.

### 9-Ethylcarbazole *(**4**)* from 10-ethylphenothiazine-5-oxide *(**7**)* using n-butyllithium

The same general procedure as above was followed except 1.6 M *n*-butyllithium (0.383 mL, 0.315 g, 4.92 mmol) was reacted with 10-ethylphenothiazine-5-oxide (**7**, 0.300 g, 1.23 mmol) [11]. After purification, 0.113 g (0.578 mmol, 47% yield) of 9-ethylcarbazole (**4**) was collected; m.p. 67-68 °C; IR and NMR identical in all respects to an authentic sample.
